# Biological Network Approach for the Identification of Regulatory Long Non-Coding RNAs Associated With Metabolic Efficiency in Cattle

**DOI:** 10.3389/fgene.2019.01130

**Published:** 2019-11-22

**Authors:** Wietje Nolte, Rosemarie Weikard, Ronald M. Brunner, Elke Albrecht, Harald M. Hammon, Antonio Reverter, Christa Kühn

**Affiliations:** ^1^Institute of Genome Biology, Leibniz Institute for Farm Animal Biology (FBN), Dummerstorf, Germany; ^2^Institute of Muscle Biology and Growth, Leibniz Institute for Farm Animal Biology (FBN), Dummerstorf, Germany; ^3^Institute of Nutritional Physiology “Oskar Kellner,” Leibniz Institute for Farm Animal Biology (FBN), Dummerstorf, Germany; ^4^Commonwealth Scientific and Industrial Research Organisation (CSIRO) Agriculture and Food, Queensland Bioscience Precinct, St Lucia, QLD, Australia; ^5^Faculty of Agricultural and Environmental Sciences, University Rostock, Rostock, Germany

**Keywords:** Bos taurus, metabolic efficiency, co-expression network analysis, long non-coding RNA, Functional Annotation of Animal Genomes

## Abstract

**Background:** Genomic regions associated with divergent livestock feed efficiency have been found predominantly outside protein coding sequences. Long non-coding RNAs (lncRNA) can modulate chromatin accessibility, gene expression and act as important metabolic regulators in mammals. By integrating phenotypic, transcriptomic, and metabolomic data with quantitative trait locus data in prioritizing co-expression network analyses, we aimed to identify and functionally characterize lncRNAs with a potential key regulatory role in metabolic efficiency in cattle.

**Materials and Methods:** Crossbred animals (n = 48) of a Charolais x Holstein F_2_-population were allocated to groups of high or low metabolic efficiency based on residual feed intake in bulls, energy corrected milk in cows and intramuscular fat content in both genders. Tissue samples from jejunum, liver, skeletal muscle and rumen were subjected to global transcriptomic analysis via stranded total RNA sequencing (RNAseq) and blood plasma samples were used for profiling of 640 metabolites. To identify lncRNAs within the indicated tissues, a project-specific transcriptome annotation was established. Subsequently, novel transcripts were categorized for potential lncRNA status, yielding a total of 7,646 predicted lncRNA transcripts belonging to 3,287 loci. A regulatory impact factor approach highlighted 92, 55, 35, and 73 lncRNAs in jejunum, liver, muscle, and rumen, respectively. Their ensuing high regulatory impact factor scores indicated a potential regulatory key function in a gene set comprising loci displaying differential expression, tissue specificity and loci overlapping with quantitative trait locus regions for residual feed intake or milk production. These were subjected to a partial correlation and information theory analysis with the prioritized gene set.

**Results and Conclusions:** Independent, significant and group-specific correlations (|r| > 0.8) were used to build a network for the high and the low metabolic efficiency group resulting in 1,522 and 1,732 nodes, respectively. Eight lncRNAs displayed a particularly high connectivity (>100 nodes). Metabolites and genes from the partial correlation and information theory networks, which each correlated significantly with the respective lncRNA, were included in an enrichment analysis indicating distinct affected pathways for the eight lncRNAs. LncRNAs associated with metabolic efficiency were classified to be functionally involved in hepatic amino acid metabolism and protein synthesis and in calcium signaling and neuronal nitric oxide synthase signaling in skeletal muscle cells.

## Introduction

In recent years the focus of livestock production and farming has shifted in developed countries towards a stronger emphasis on resource efficiency and sustainability (Thornton, 2010). In cattle, energy metabolism, nutrient conversion and efficient use of primary resources are of increasing economic and ecological importance to breeders and consumers. Genomic selection and the use of biomarkers greatly facilitate the improvement of complex phenotypes, e.g. feed efficiency, which remain cost- and time-consuming to measure (Kenny et al., 2018).

Some pivotal gene mutations are known in major livestock production traits, e.g. a meta-analysis on stature in cattle identified *PLAG1* as a major regulator and pointed towards putative causal mutations (Bouwman et al., 2018). In pigs, the scavenger receptor cysteine-rich domain 5 in gene *CD163*, when not being translated, led to resistance to porcine reproductive and respiratory syndrome virus 1 infection (Burkard et al., 2018). Pigs that did not express the receptor protein were susceptible to the infection. For the region between *LCORL* and *NCAPG*, which has been associated with growth or feed efficiency in a number of species (cattle, horse, human), multiple mappings have narrowed down the region of interest but the causal mutation remains unknown (Widmann et al., 2015;Bouwman et al., 2018). A large part of the variation in traits like feed efficiency, growth and carcass traits remains still unexplained (Hardie et al., 2017; Medeiros de Oliveira Silva et al., 2017; Seabury et al., 2017) and genome-wide association studies repeatedly pointed towards quantitative trait loci (QTL) outside protein-coding genes (Ibeagha-Awemu et al., 2016; Seabury et al., 2017;Higgins et al., 2018).

Due to their gene expression regulatory potential, long non-coding RNAs (lncRNAs) have emerged as potential key regulators for diverse biological processes, such as X-chromosomal inactivation and dosage compensation (Brown et al., 1992; Clemson et al., 1996), vernalization/ flowering in plants (Csorba et al., 2014), as well as human cancer biology as reviewed by Serviss et al. (2014).

Recently, lncRNAs have been suggested as therapeutic targets for diabetes and other metabolic diseases because of their involvement in lipid metabolism, adipogenesis and fat deposition (Chen et al., 2018a; Liu et al., 2018;Zeng et al., 2018). In mammals, lncRNAs were further identified as key regulators of energy metabolism and lipogenesis (Yang et al., 2016). In adipocytes, these genomic elements also play an integral part in the insulin-signaling pathway (Degirmenci et al., 2019). A central regulatory role of lncRNAs was furthermore observed in skeletal muscle in myogenesis and muscle cell differentiation: *SYISL* has been shown to regulate myoblast proliferation and fusion and acts in an inhibitory way in myogenic differentiation (Jin et al., 2018), *Irm* enhances myogenic differentiation during myogenesis through the binding to *MEF2D* (Sui et al., 2019), and *lnc-mg* overexpression has directly been linked to muscle hypertrophy in mice, whereas a knock-out led to dystrophy (Zhu et al., 2017). It is likely that lncRNAs contribute significantly to economically important production traits and divergent phenotypes in livestock as well. Since they show little sequence conservation across species and their expression appears to be mainly species specific and spatiotemporal (Ulitsky et al., 2011;Ulitsky and Bartel, 2013), knowledge transfer remains a challenging issue. The identification and functional characterization of lncRNAs needs to be performed for each species, and this fits into one of the major goals of the consortium for the Functional Annotation of Animal Genomes (FAANG, www.animalgenome.org/community/FAANG/)that strives to identify and annotate functionally relevant elements in livestock genomes.

Another key feature of lncRNAs is their low expression level compared to protein-coding genes (Derrien et al., 2012), which makes their detection challenging. From transcription factors it is known, that little changes in abundance can however have tremendous consequences if these have high regulatory potential in terms of gene expression (Vaquerizas et al., 2009) and we postulated an analogous phenomenon for lncRNAs. For instance, the knockout of the lowly expressed lncRNA *ßlinc* in mice impaired the correct formation of pancreatic islets and severely changed the glucose homeostasis in adult animals (Arnes et al., 2016). A low and tightly regulated gene expression has implications for differential expression (DE) analyses, because little changes in expression are often not recognized as significant due to lack of power in standard experimental designs. Therefore, other approaches are necessary when aiming to identify and functionally annotate key regulatory lncRNAs. A tested and proven method in the screening for critical transcription factors from gene expression data, which are typically low in abundance but have high regulatory power as reviewed by Vaquerizas et al. (2009), is network co-expression analysis that incorporates the regulatory impact factor (RIF) metrics and a partial correlation and information theory (PCIT) (Reverter et al., 2010; Perez-Montarelo et al., 2012). This approach has previously also led to the identification of regulatory elements associated with puberty (Canovas et al., 2014; Nguyen et al., 2018) and feed efficiency in cattle (Alexandre et al., 2019). We assumed that this rational network approach could also be used as a hypothetical generation tool for the systematic detection of lncRNAs with important regulatory potential.

In this study, we took advantage of a unique F_2_ cross-population of meat and dairy cattle breeds (Charolais x Holstein) (Kühn et al., 2002) that has been deeply phenotyped and genotyped.

Earlier studies have shown that in this cross population a gene variant of the *NCAPG* gene is associated with fetal and pubertal growth (Eberlein et al., 2009; Weikard et al., 2010). By integrating quantitative metabolite data with genotype information, this *NCAPG* genotype was found to be associated with plasma arginine levels (Weikard et al., 2010). A systems biology approach, which combined metabolome data, growth-associated phenotypic and genetic information, revealed a functional gene interaction network characterizing the intensive growth phase at the beginning of the pubertal growth interval (Widmann et al., 2013). Potential interaction partners of the *NCAPG* gene were predicted and the functional role of the *NCAPG* gene as a growth regulator linked to the arginine NO metabolism was concluded. A combined phenotype–metabolome–genome analysis was also used to identify genetic switches of associated molecular signaling pathways linked to variance in efficiency of feed conversion (Widmann et al., 2015).

This current study on the regulatory role of lncRNAs for metabolic efficiency was aimed to contribute to a more detailed elucidation of the molecular background of this complex physiological trait and help to characterize divergent metabolic types with respect to nutrient partitioning. Therefore, phenotypic information, transcriptomic data from four metabolically relevant tissues and QTL information were used to establish a prioritized gene set that was submitted to the combinational RIF metrics and subsequently to the PCIT algorithm for co-expression network creation. The integration of metabolomic profiles through correlation with transcriptomic data added valuable information for the interpretation of biological functions.

## Materials and Methods

### Design of the Study

For this study, we made use of 48 animals (24 bulls, 24 cows) of a F_2_-population [SEGFAM (Kühn et al., 2002)] from a Charolais × Holstein cross. The cross population was bred at the Leibniz Institute for Farm Animal Biology in Dummerstorf (Germany) and kept under standardized housing and feeding conditions as previously described (Eberlein et al., 2009; Weikard et al., 2010;Widmann et al., 2011). Males were slaughtered at 18 months of age and females were slaughtered after their second parity at 30 days postpartum. Based on residual feed intake (RFI) in bulls and energy corrected milk yield (ECM_w_) in cows as well as intramuscular fat content (IMF) of *M. longissimus dorsi* in both genders, animals were assigned to either of the two groups: high or low metabolic efficiency (**Table 1**). In this study we defined high metabolic efficiency in cattle as the preference to accrete or secrete protein while receiving the same diet as their inefficient conspecifics, which were characterized by a clear tendency to accrete fat instead of protein. In European production systems, those animals are most sustainable and economically efficient producers, which build up protein mass (muscle) with little fat content or, in case of females, secrete high amounts of milk.

**Table 1:** Sample characteristics.

Metabolic efficiency group	Number of animals	Sex	RFI^1^ in last month of life (bulls)	ECM^2^_w_ (cows)	IMF^3^ (both sexes)	CFC^4^ (both sexes)
			µ^5^ (SD^6^)	µ (SD)	µ (SD)	µ (SD)
**High**	25	12 males 13 females	-21.30 (4.44)	190.87 (22.02)	3.46 (1.30)	15.93 (3.16)
**Low**	23	12 males 11 females	20.83 (4.41)	30.97 (9.18)	5.51 (2.34)	22.93 (4.88)

^1^RFI, residual feed intake; ^2^ECM_w_, energy corrected milk 7 days before slaughter; ^3^IMF, intramuscular fat content (given in percent, measured in M. longissimus dorsi); ^4^CFC, carcass fat content; ^5^µ, mean; ^6^SD, standard deviation.

Cows were categorized as highly efficient if their milk yield within the 7 days prior to slaughter was above 140 kg energy correct milk (ECM_w_) and the carcass fat content (CFC) was less than the average CFC of all cows plus one standard deviation. In contrast, cows were classified as lowly efficient if their milk yield within the last week was between 14 and 40 kg ECM_w_ and the CFC was above the average CFC of all cows minus one standard deviation. For all cows, the calving interval had to be less than 540 days, the maximum age was 1,510 days and they had to be free of pathological findings with metabolic implications noted after slaughter. Cows that were categorized as highly efficient (high ECM_w_) on average had a lower CFC (mean 17.1%, SD 2.7%) and lowly efficient cows (low ECM_w_) had a higher CFC (mean 25.9%, SD 3.6%) than the mean of the population (21.8%, SD 5.3%, n = 242). In addition, highly efficient cows had a lower IMF (mean 4.16%, SD 1.60%) and the lowly efficient cows had a higher IMF (mean 6.46%, SD 2.53%) than the mean of the population (5.21%, SD 2.21%, n = 242).

The individual milk volume yield per cow was measured on a daily basis and the milk composition was determined once per week. The trait included in cow selection for this study corresponded to the weekly ECM determined for the 7 days before slaughter (ECM_w_). The formula presented by Kirchgeßner (1997) was modified accordingly for the one week interval (F% = milk fat percentage, P% = milk protein percentage):

$$ECM_{w}=\frac{0.37\;F\%+0.21\;P\%+0.95}{3.1}\times MY-7d$$

cows, the ECM_w_ was used as a substitute feature for feed efficiency, because the facilities did not allow for RFI measurement in cows during the time of the experiment.

For bulls, the decisive factor for animal selection was RFI calculated for the last month prior to slaughter. The RFI equals the animals' energy intake while considering the average daily gain and metabolic mid-weight (average body weight of months of life 17 to 18 raised to the power of 0.75) (Archer et al., 1997).

Bulls with a low RFI (at least 1 standard deviation below average) were assigned to the high metabolic efficiency group and bulls with a high RFI (at least one standard deviation above average) were assigned to the low metabolic efficiency group. In their last month of life, all bulls had to have a positive daily weight gain and no less than the population average minus one standard deviation. Bulls that were categorized as highly efficient (negative RFI) on average had a lower CFC (mean 14.2%, SD 3.0%) and lowly efficient bulls (positive RFI) had a higher CFC (mean 20.2%, SD 4.4%) than the population mean (mean 16.5%, SD 4.0%, n = 246). Analogously to cows, highly efficient bulls had a lower IMF (mean 1.71%, SD 1.00%) and the lowly efficient bulls had a higher IMF (mean 4.64%, SD 1.84%) than the population mean (mean 3.67%, SD 1.76%, n = 246).

### Plasma Metabolic Profiles

Blood samples were collected from all individuals (n = 48) at slaughter. Plasma samples were sent to Metabolon Inc. (Durham/NC, USA) for the establishment of holistic metabolite profiles that included 640 biochemical compounds and molecules. Metabolites with more than five animals with missing data were excluded. After this filtering step, 490 metabolites remained and missing values were imputed with the minimum measurement, assuming that missing values were due to concentrations below the detection limit. Values were then scaled without centering for each metabolite in R (Core Team 2018) with the scale-function.

All experimental procedures were carried out according to the German animal care guidelines and were approved and supervised by the relevant authorities of the State Mecklenburg-Vorpommern, Germany (State Office for Agriculture, Food Safety and Fishery; LALLF M-V/TSD/7221.3-2.1-010/03).

### Sampling, RNA Isolation, Library Preparation, and Sequencing

Tissue samples were collected from jejunum mucosa, liver (*Lobus caudatus*), skeletal muscle (*M. longissimus dorsi*), and rumen (*Saccus ventralis*, papillary base) directly after slaughtering and dissection, shock frozen in liquid nitrogen and subsequently stored at -80°C.

For RNA extraction from muscle and rumen, frozen samples (100 mg) were treated with 1 ml TRIzol reagent (Invitrogen, Darmstadt, Germany) and subjected to the Precellys-24 homogenizer (5,500 rpm, 2 × 15 s, lysing kit containing 1.4 mm ceramic beads). For RNA extraction from liver and jejunum, frozen tissue samples were grinded in liquid nitrogen and 30 mg were used for further purification steps. No TRIzol was used for liver and jejunum samples. All samples were then subjected to an on-column-purification step with the NucleoSpin RNA II kit (Macherey & Nagel, Düren, Germany) including a DNase digestion to remove genomic DNA. In addition, the RNA was tested for remaining traces of DNA contamination and, in case of remaining DNA residues, further cleansed according to Weikard et al. (2012).

The RNA concentration and integrity were measured with a Qubit Fluorometer (Invitrogen, Germany) and a 2100 Bioanalyzer Instrument (Agilent Technologies, Germany). Stranded, ribodepleted and indexed libraries were prepared from 1 µg total RNA using the TruSeq Stranded Total RNA Ribo-Zero H/M/R Gold Kit (Illumina, San Diego, USA) and subjected to paired-end sequencing (2 × 100 bp) in a multiplexed design on a HiSeq 2500 Sequencing System (Illumina).

### Alignment and Assembly

After quality control of raw sequencing reads with FastQC (Andrew, 2010), adapter and quality trimming were performed with Cutadapt v. 1.16 (Martin, 2011) and Quality Trim v. 1.6.0 (Robinson, 2015), respectively. In Quality Trim the start of sequences was also trimmed (option -s) and the maximum number of N bases was set to 3, while the minimum base quality was set to 15. Reads were then mapped in a guided alignment with HISAT2 v.2.1.0 (Kim et al., 2015) to the bovine reference genome UMD.3.1 [Ensembl annotation release 92 (Frankish et al., 2017)]. After sorting and indexing of BAM files with samtools v.1.6 (Li et al., 2009), samples were individually assembled with Stringtie v.1.3.4d (Pertea et al., 2015) based on the reference genome and annotation used for alignment. Using the individually assembled samples (n = 204) from all four tissues and the bovine reference genome, we built a new merged annotation in Stringtie across tissues, while specifying for minimal transcript coverage across samples of 15 read alignments per exonic base. In addition to the 192 samples (48 animals, four tissues) included in the subsequent steps for DE and network analyses, we also took benefit from rumen, liver and muscle samples of further four individuals from the same experimental herd. These samples were subjected to exactly the same processing steps as the 192. The new merged annotation was used for fragment counting with featureCounts (subread v.1.6.1) (Liao et al., 2014), while allowing for fractional counting and specifying for reverse strandedness.

### Long Non-Coding RNA Prediction and Fragment Counting

LncRNAs were identified *in-situ* with FEELnc (Wucher et al., 2017), a bioinformatics tool for lncRNA prediction and annotation, using the merged transcript annotation and the bovine reference genome and annotation UMD3.1 release 92. FEELnc excludes transcripts annotated as protein coding and subsequently keeps transcripts with a minimum length of 200 nt and at least two exons and only monoexonic transcripts with antisense localization. Other monoexonic transcripts were excluded to reduce the number of false positives, which might arise from the mapping of repetitive sequences (Wucher et al., 2017), DNA contamination (Haerty and Ponting, 2015) and in general transcriptional noise (Kern et al., 2018). For those transcripts matching the requirements, the coding potential of remaining transcripts was determined in shuffling mode.

### Fragment Count Normalization

For further pipeline steps, except for the DE analysis, fragments per kilobase million (FPKM) were calculated from the featureCounts derived fragment counts. Genes were filtered for a minimal average expression value of 0.2 FPKM in at least one of the four tissues and ribosomal and spliceosomal RNA genes were excluded (Metazoan signal recognition particle RNA, U6 spliceosomal RNA, small nucleolar RNA U6-53). For further analyses of FPKM values performed in this study, a log2-scale of the data was used (for log transformation a pseudo-count of 0.001 was added).

### Prioritized Gene List

Gene co-expression networks are a useful tool when trying to deduce the potential biological function of genes, novel loci and non-coding elements (van Dam et al., 2017), assuming the guilt-by-association principle. In order to create meaningful networks that have a targeted focus on our phenotype (metabolic efficiency), we created a set of prioritized genes where genes had to belong to at least one of these four categories: differentially expressed (DE) genes in at least one of the four investigated tissues, tissue-specific (TS) genes, genes harboring a QTL for milk production or RFI (QTL) according to the literature, and predicted lncRNAs. Small nucleolar RNAs (snoRNAs), ribosomal RNAs, spliceosomal RNAs, and Y-RNAs were excluded from the set.

#### Differential Expression Analysis

A DE analysis for the high and low metabolic efficiency group was performed within tissues and across sexes in R with the package DEseq2 (Love et al., 2014). Fragment counts from featureCounts were used as input and normalization was performed within DEseq2. To exclude very lowly expressed transcripts within a tissue, the minimal fragment count threshold was set to at least 10 fragments for 10 out of 48 individuals. Ribosomal genes were excluded from the analysis and year of slaughter and sex were used as factors in the model. The significance threshold was set to q < 0.05 [Benjamini–Hochberg (BH) test].

#### Tissue Enriched Genes

The expression (log2-transformed FPKM) of a gene was defined as enriched in a particular tissue, if the abundance in the other three tissues was less than half the average across all tissues and above the average plus one standard deviation in the tissue at hand. Throughout the further course of this study, we refer to these genes as TS.

#### Genes Harboring a Quantitative Trait Locus

We extracted QTL for milk production traits (MY) and RFI in cattle from the Animal QTL database (Park et al., 2018) and then screened our dataset in Ensembl Biomart (http://asia.ensembl.org/biomart/martview, accession date 28 March 2019) for genes that overlapped with these QTL regions. A physical overlap of the QTL and the gene is needed for a gene hit, while close neighborhood is not sufficient.

### Regulatory Impact Factor Analysis

The RIF (Reverter et al., 2010) analysis makes use of two alternative metrics (RIF1 and RIF2) that attribute scores to potential key regulators. The strength of the score depends on the change in correlation between the regulator and its target in two groups or treatments, the level of DE of the target gene, and the general expression level of the target gene. We conducted RIF analyses within tissues and across metabolic efficiency groups to assess the regulatory capacity of lncRNAs in a set of prioritized genes (lncRNA, DE, TS, QTL harboring). Therefore, RIF metrics were calculated within each tissue for a prioritized gene set (including log2(FPKM) data) that comprised genes which were DE or TS in that tissue, harbored a QTL or were characterized as a lncRNA. Naturally, some of the QTL-genes might have zero expression in one or more of the tissues. To prevent erroneously high RIF scores stemming from low variation in gene expression, an additional filter for expression level was applied (on top of minimal average expression of 0.2 FPKM in at least one tissue). Only genes with abundance above tissue average were kept for the RIF analysis.

A high RIF1 score was assigned to lncRNAs that were consistently co-expressed with abundant target genes in both metabolic efficiency groups. A high RIF2 score was attributed to lncRNAs that displayed the most altered ability to predict the abundance of target genes between groups, meaning that a lncRNA exhibited strong correlation to a target on one condition but none or a reverse correlation in the other. RIF scores were standardized with a z-score. Key regulators (lncRNA) were considered of significant importance and were included in further analyses if they had an absolute RIF1 or RIF2 z-score of ≥1.96, meaning that these lncRNAs and their scores were outside the 95% confidence interval, corresponding to a significance level of p = 0.05 in a t-test.

### Partial Correlation and Information Theory

The PCIT (Reverter and Chan, 2008) tests for significant pairwise correlations between two elements while accounting for all possible three-way combinations in the dataset that include either of the pair elements. Importantly, the PCIT recognizes independent, significant correlations regardless of the strength of correlation. Within the high and low metabolic efficiency groups, the PCIT approach across all tissues was used to investigate for independent correlations of lncRNAs that had significant RIF scores with DE genes, TS genes, and QTL harboring genes.

Results were filtered for significant correlations (minimal correlation strength |r| > 0.8) between a lncRNA and another gene that were exclusive for the high or low metabolic efficiency group, meaning that the correlation was significant in one group but not in the other. The visualization was realized in Cytoscape 3.6.1 (Shannon et al., 2003).

### Characterization of Key Regulatory Long Non-Coding RNAs

#### Blast Search Against New Bovine Assembly

Highly connected lncRNAs with more than 100 directly linked nodes (genes) were selected from each network for further scrutiny. Since the prediction of lncRNAs was based on a merged annotation, which was reference guided by UMD3.1, Ensembl release 92, we wanted to investigate the sequence homology and annotation status of key lncRNAs in the new bovine assembly ARS1.2 annotated in Ensembl release 95. The lncRNA sequences were blasted online with the blastn suite using the MegaBlast algorithm, specifying for high sequence similarity and otherwise default parameters (Altschul et al., 1990) (https://blast.ncbi.nlm.nih.gov/Blast.cgi, accessed Mai 2019) against the new bovine assembly (ARS-UCD1.2, www.ncbi.nlm.nih.gov/assembly/GCA_002263795.2; GenBank accession NKLS00000000.2; www.ensembl.org/Bos_taurus/Info/Index). We considered blast hits to indicate high homology if the sequence identity was at least 98% in a region covering at least 200 nucleotides.

#### Pathway Enrichment Analysis

**Figure 1:**
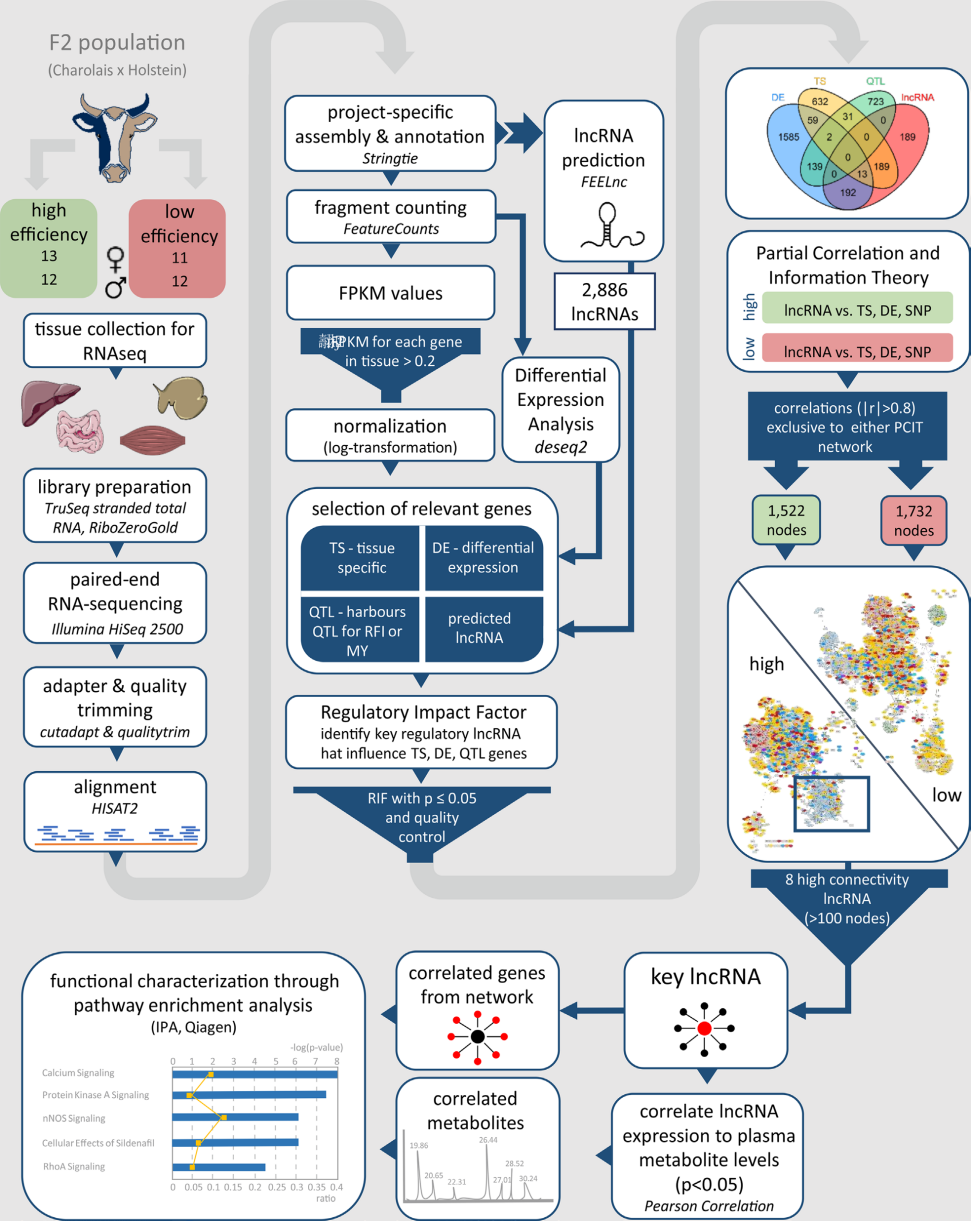
Workflow for the identification and functional characterization of key lncRNAs with regulatory potential in two contrasting biological conditions. The phenotypes under investigation were high and low metabolic efficiency in a Charolais x Holstein cross-population. lncRNA, long non-coding RNA; FPKM, fragments per kilobase transcript length per million reads; TS, tissue specific; DE, differentially expressed; QTL, quantitative trait locus; RFI, residual feed intake; MY, milk production; RIF, regulatory impact factor; PCIT, partial correlation and information theory.

To assess the possible biological function of high connectivity lncRNAs, we performed a pathway enrichment analysis based on genes identified as correlated (|r| > 0.8) in the PCIT analyses and also including blood plasma metabolites that were significantly (p ≤ 0.05) correlated with the high connectivity lncRNAs. To this end, a pairwise Pearson correlation analysis between blood-plasma metabolites and lncRNA expression in the tissue, where the lncRNA was most abundant, was performed in R with the function rcorr of the Hmisc package (Harrell and Frank, 2019). The list of significantly correlated metabolites (p ≤ 0.05) and genes (adjacent network nodes with |r| > 0.8) were analysed using the Ingenuity Pathway Analysis (IPA: QIAGEN Inc., www.qiagenbioinformatics.com/products/ingenuity-pathway-analysis) (Kramer et al., 2014). The workflow from group formation and tissue sampling up to the functional characterization of key lncRNAs is visualized for better comprehensibility and clarity in **Figure 1**.

## Results

### RNA Preparation, Sequencing, Alignment, and Mapping

The average RNA integrity (RIN) across the four tissues was 8.22 ± 0.81 (**Table 2**). After quality trimming the average RNA sequencing depth was at 48 million read pairs per sample. A total of 9 out of 192 samples reached less than a 40 million read pair coverage. The alignment of reads with HISAT2 to the bovine reference genome UMD.3.1 (Ensembl release 92) resulted in an average alignment rate of 92.98 ± 9.50%. Compared with the other tissues, rumen scored a distinctly lower rate (78.00 ± 7.75%). The average mapping rate across all samples to the customized annotation, which contained 30,072 loci, was 81.89%. The tissue specific average mapping rate was lowest in rumen, of comparable dimension in jejunum and muscle, and highest in liver.

**Table 2:** Overall and tissue-specific RNA sequencing, alignment, and mapping statistics.

	RIN^1^		Sequencing depth [read pairs]		Alignment to UMD.3.1 [%]		Mapping to project-specific annotation [%]	
	µ^2^	SD^3^	µ	SD	µ	SD	µ	SD
**All**	8.22	0.81	48,041,209	5,601,638	92.98	9.50	81.89	8.67
**Jejunum**	8.73	0.44	48,954,376	3,993,201	96.91	0.31	84.99	2.20
**Liver**	8.00	0.62	50,093,826	5,869,833	98.43	0.20	91.36	1.21
**Muscle**	7.55	0.85	47,117,156	5,815,843	98.59	0.13	82.42	1.79
**Rumen**	8.41	0.86	45,999,477	5,587,407	78.00	7.75	69.05	4.67

^1^RIN, RNA integrity number, ^2^µ, mean, ^3^SD, standard deviation.

### Long Non-Coding RNA Prediction

Based on the merged annotation, FEELnc predicted 26,740 mRNAs and 7,646 lncRNA transcripts (3,287 loci), out of which 544 were without potential positional interaction partner gene within the default window size of 10,000 to 100,000 nucleotides. Those 7,102 lncRNA transcripts with an assigned potential positional interaction partner were generated by 3,051 loci (**Table 3**, **Supplementary Table 1**). FEELnc distinguishes between intergenic and genic lncRNA with different subtypes (see Wucher et al. (2017) for a graphical explanation). LncRNAs are also classified according to their position to neighboring protein coding genes (interaction partner gene). For intergenic lncRNAs, the best partner gene is closest in terms of distance in base pairs and for genic lncRNAs the best partner gene directly overlaps with it, preferably at an exon. All predicted 7,646 lncRNA transcripts were considered for further computational analyses.

**Table 3:** Characterization of high connectivity long non-coding RNAs from networks specific for high or low metabolic efficiency in cattle.

Identifier		FEElnc Prediction (based on UMD3.1 release 92)			FPKM	Position & Structure					Differential Expression Analysis			
MSTRG	TN	Closest partner gene (gene symbol)	Direction, type, location	Distance bp	Mean	BTA	Exons	Start bp	Strand	Exonic length	Log2 FC	p-value	q-value (BH)	Tissue
MSTRG.4740	1	ENSBTAG00000002062 (TRPA1)	Anti-sense, genic, intronic	0	6.74	14	2	37760114	+	437	0.77	2.10E-04	9.13E-03	Liver
MSTRG.4926	1	ENSBTAG00000021964 (CDH17)	Anti-sense, genic, exonic	0	0.78	14	18	72437085	-	3,321	NA	NA	NA	None
MSTRG.9051	1	ENSBTAG00000004651 (NME1)	Anti-sense, intergenic, downstream	358	2.57	19	2	36225984	-	1,866	NA	NA	NA	None
	2			1,587				36227213						
MSTRG.10337	1	ENSBTAG00000005353 (DES)	Anti-sense, genic, exonic	0	5.37	2	9	108075380	-	2,989	NA	NA	NA	None
MSTRG.17681	1	ENSBTAG00000005726 (HNRNPA2B1)	Anti-sense, intergenic, upstream	9,170	19.80	4	3	70184682	-	1,552	-0.38	8.45E-05	5.04E-03	Liver
MSTRG.18433	1	ENSBTAG00000015828 (FKBP11)	Sense, intergenic, upstream	5,808	8.94	5	2	31038376	+	2,683	NA	NA	NA	None
MSTRG.19098	1	ENSBTAG00000046324 (C-type lectin domain family 2 member D11)	Anti-sense, genic, exonic	0	1.72	5	5	100926473	+	1,570	-0.63	1.27E-06	3.04E-04	Liver
MSTRG.19312	1	ENSBTAG00000009886 (KDELR3 )	Anti-sense, genic, exonic	0	25.84	5	3	110665971	-	5,768	NA	NA	NA	None
	3							110670286						

*MSTRG, identifier from project-specific bovine transcriptome annotation; TN, transcript number; FPKM, fragments per kilobase million; BTA, bovine chromosome, bp, base pair; FC, foldchange; BH, Benjamini–Hochberg, NA, not available.

The total of 3,287 lncRNA loci are equally distributed in terms of strandedness (50.6% on the plus strand, 49.41% on the minus strand), and in a locus-based approach (considering the transcript with highest exon number for each locus) the median number of exons per transcript was 3 (average number of exons per transcript: 4.9 ± 8.2). The total exon length geometric mean of the lncRNA loci amounted to 2,201.0 bp.

### Prioritized Gene List for Co-Expression Analysis

After filtering the 30,072 genes in the merged annotation for minimal expression (average FPKM across all samples >0.2 in at least one tissue) and exclusion of ribosomal and spliceosomal RNA genes, the dataset contained 22,625 genes out of which 2,886 were lncRNAs, meaning that 401 lncRNAs were removed from RIF and subsequent PCIT co-expression analysis due to very low abundance.

#### Differential Expression Analysis

**Figure 2:**
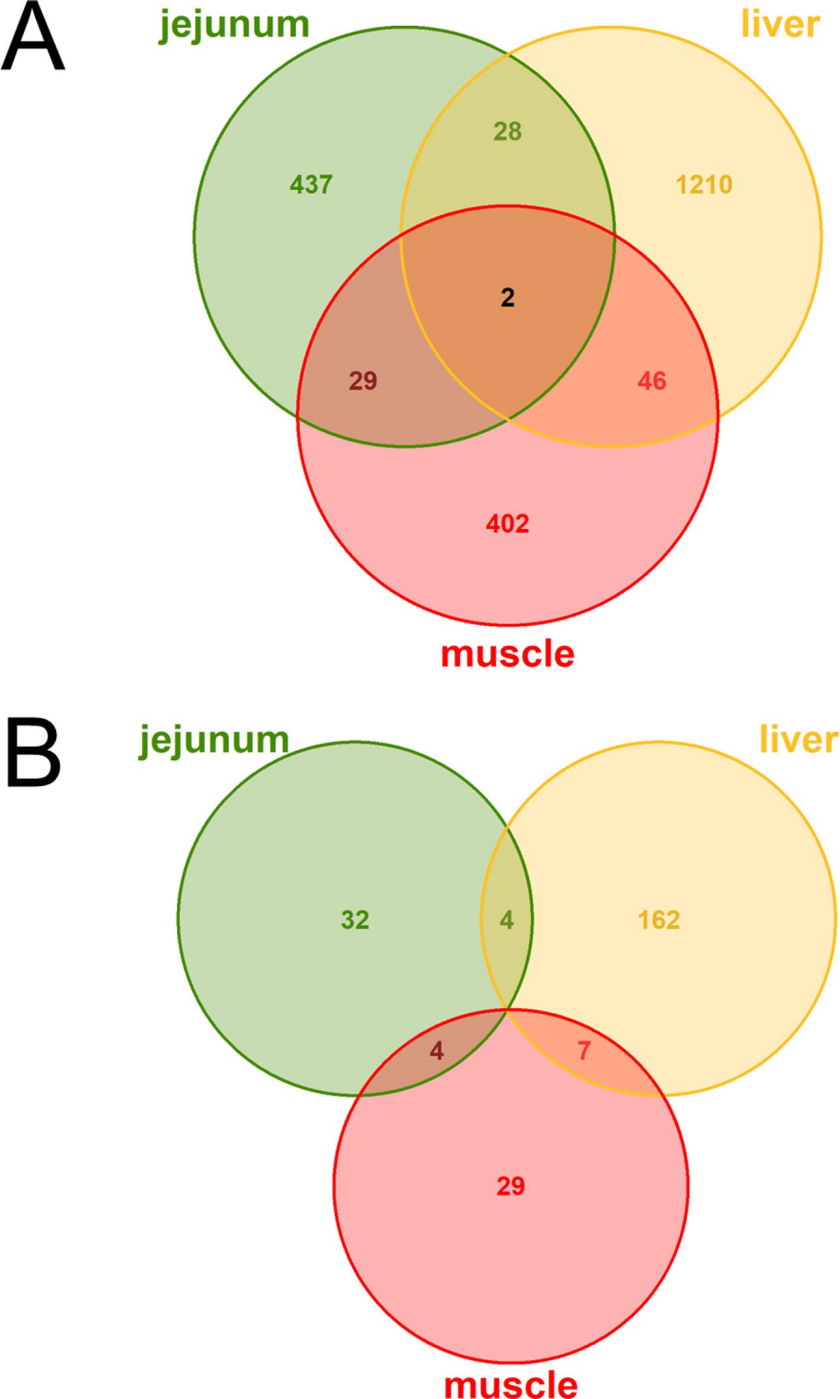
Venn diagrams of **(A)** all loci **(B)** exclusively lncRNAs with differential expression (DE) between high and low metabolic efficiency in cattle. DE analysis was performed within the tissues jejunum, liver, muscle, and rumen. No loci were significantly [q-value (Benjamini–Hochberg) < 0.05] DE in rumen.

The DE analysis yielded a total of 2,154 unique significantly (q < 0.05) DE genes between the high and low metabolic efficiency group with 496 DE genes in jejunum, 1,286 DE genes in liver, 479 DE genes in muscle, and no significant differences in rumen (**Figure 2A**). Generally, we observed little overlap of differentially expressed loci between tissues. Out of these unique 2,154 DE genes, 238 were predicted to be lncRNAs corresponding to 11.05%. We observed 40 DE lncRNAs in jejunum, 173 DE lncRNAs in liver, 40 DE lncRNAs in muscle, and none in rumen (**Figure 2B**).

#### Tissue Enriched Genes

We found a total of 930 genes to be tissue-specifically expressed out of the 22,625 genes, which had passed the initial minimal expression threshold (average expression > 0.2 FPKM in at least on tissue). Out of those 930 genes, 279 were TS in jejunum, 283 in liver, 204 in muscle, and 164 in rumen. Thereof, 21.9% were lncRNAs with 42 in jejunum, 65 in liver, 48 in muscle, and 49 in rumen.

#### Quantitative Trait Locus Harboring Genes

The database AnimalQTL listed 278 QTL for RFI and 1,881 QTL for milk production traits, which were distributed across 1,615 genes out of which 1,064 passed the minimal expression threshold (average expression > 0.2 FPKM in at least one tissue) in our dataset.

### Regulatory Impact Factor to Select Long Non-Coding RNAs With a Potential Regulatory Effect on Metabolic Efficiency

The input prioritized gene lists filtered for expression level for the tissue specific RIF analysis contained 2,097 loci for jejunum (880 lncRNAs), 1,890 loci for liver (614 lncRNAs), 961 loci for muscle (363 lncRNAs), and 1,458 loci for rumen (755 lncRNAs). RIF scores were then calculated for the lncRNAs in these gene sets.

With a significance threshold of a RIF1 or RIF2 score ≥ 1.96, the tissue specific RIF analyses identified 92 potential key lncRNAs in jejunum, 55 in liver, 35 in muscle, and 73 in rumen. In total 240 unique lncRNAs had a RIF score ≥ 1.96 in at least one tissue and were considered for subsequent PCIT analysis.

### Partial Correlation and Information Theory Approach to Identify Long Non-Coding RNA-Associated Co-Expression Networks

**Figure 3:**
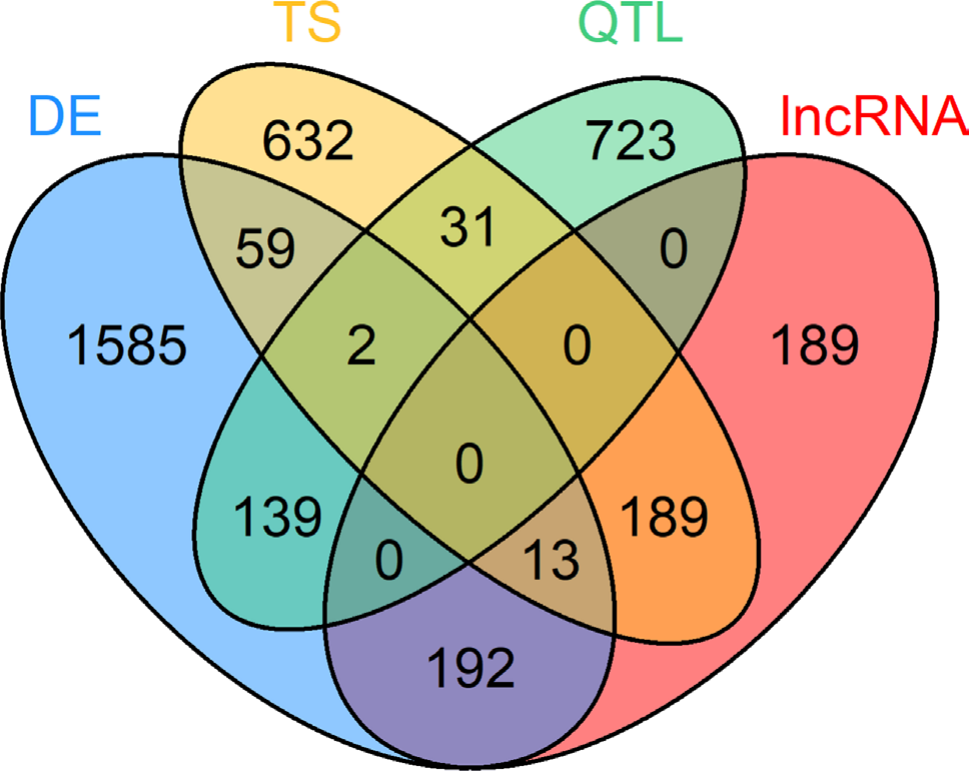
Venn diagram of 3,754 loci selected for co-expression network construction. Loci belonging to at least one of these four categories were considered: differential expression (DE) in at least one tissue, tissue specific (TS) expression, harboring a QTL for residual feed intake and or milk production (QTL) and key regulatory long non-coding (lnc) RNAs [significant (p < 0.05) regulatory impact factor score].

For the within-tissue RIF analysis, the sets of DE genes, TS genes, QTL harboring genes and lncRNAs had been filtered for a seizable expression level (abundance above average expression in the respective tissue) to facilitate a reliable calculation of correlation. For the PCIT analysis, a similar filter for minimal expression was applied: abundance above average expression across all samples in at least one tissue when combining DE genes and TS genes from all tissues with the QTL genes and lncRNAs with significant RIF scores. A total of 295 of the 4,049 prioritized loci were excluded due to not meeting this expression limit. The set of prioritized genes that was used for the final PCIT network analysis contained 3,754 unique genes in total. Thereof, 1,990 were DE genes, 895 QTL containing genes, 926 TS genes, and 583 lncRNAs, though some genes belonged to several categories (**Figure 3**, **Supplementary Table 2**).

The PCIT analysis was performed across tissues and results were filtered for significant correlations with a correlation strength |r| ≥ 0.8, between a lncRNA with significant RIF score and all genes from the prioritized gene list already used for RIF calculation. Furthermore, correlations had to be exclusive to either the high or low metabolic efficiency group. The high and low network contained 1,522 and 1,732 nodes (genes) respectively (**Supplementary Figure 1**, **Supplementary Figure 2**, **Supplementary Table 3**). Six and two lncRNAs showed a high connectivity (>100 nodes) exclusively in one of the two networks, which represent high and low metabolic efficiency, respectively. Thus, these eight lncRNAs stand out as potential regulatory keys for lncRNAs with respect to metabolic efficiency.

### Characterization of Key Regulatory Long Non-Coding RNAs in the Networks

#### Blast Against New Bovine Assembly

The eight lncRNAs characterized by high connectivity for high and low metabolic efficiency in the PCIT analysis were blasted against the new bovine assembly and annotation [ARS-UCD.1.2, National Center for Biotechnology Information (NCBI) release 106] (**Table 4**). If lncRNAs completely overlapped with annotated genes, the respective lncRNA was located on the opposite strand to the annotated gene (e.g. MSTRG.4926 overlapped with *CDH17* on the opposite strand). None of the eight lncRNA loci had yet been annotated as non-coding in the NCBI or the Ensembl genome annotation (ARS-UCD1.2, release 95).

**Table 4:** BLAST results for eight high connectivity long non-coding RNAs (>100 nodes) in partial correlation and information theory networks with connections exclusive for high or low metabolic efficiency.

lncRNA		BLAST against bovine reference genome (ARS-UCD1.2, release 106)				
Identifier	Network (connectivity in nodes)	Annotated gene with highest sequence homology	Identity [%]	Query cover [%]	E-Value	Position of lncRNA relative to homologous gene in ARS-UCD1.2
MSTRG.4740	Low (147)	mRNA-transient receptor potential cation channel subfamily A member 1 (TRPA1)	100.00	100.00	9.00E-116	Intronic, anti-sense
		ADP-ribosylation factor 4 (ARF4)	98.57	91.00	3.00E-100	Exonic, sense
MSTRG.4926	High (144)	Cadherin-17 precursor (CDH17)	100.00	100.00	0.00E+00	Anti-sense
MSTRG.9051	High (170)	Nucleoside diphosphate kinase A 1 isoform X1 (NME1)	99.72	100.00	0.00E+00	Sense, genic
MSTRG.10337	Low (239)	Desmin (DES)	99.93	100.00	0.00E+00	Exonic, anti-sense
MSTRG.17681	High (120)	39,201 bp at 5' side: alpha-aminoadipic semialdehyde synthase, mitochondrial precursor 88559 bp at 3' side: fez family zinc finger protein 1	98.40	99.00	0.00E+00	Sense, genic
		Chromobox protein homolog 3 isoform X1 (CBX3)	99.00	89.00	0.00E+00	Sense, genic
MSTRG.18433	High (268)	364 bp at 5' side: ADP-ribosylation factor 3; 37831 bp at 3' side: peptidyl-prolyl cis-trans isomerase FKBP11 precursor	99.96	100.00	0.00E+00	Sense, intergenic
MSTRG.19098	High (184)	C-type lectin domain family 2 member D11	100.00	100.00	0.00E+00	Anti-sense, genic
MSTRG.19312	High (212)	ER lumen protein-retaining receptor 3 (KDELR3)	100.00	99.00	0.00E+00	Anti-sense, genic

#### Pathway Enrichment Analysis

The Pearson correlation analysis between blood plasma metabolites and lncRNA expression, which was calculated prior to the pathway enrichment analysis, showed that the eight key lncRNAs were significantly (p < 0.05) correlated to very different numbers of metabolites. Correlations ranged from one (MSTRG.18433) to 117 (MSTRG.4740) metabolites, out of which an average of 75% was successfully mapped in the IPA database and used in the subsequent enrichment analyses (**Supplementary Table 4**). The correlation strength ranged from -0.53 to + 0.48 with an average of |0.35|.

Pathway enrichment analysis for each of the eight key lncRNAs with their respective correlated metabolites and genes showed that calcium signaling was the most strongly enriched canonical pathway for half of the key lncRNAs (MSTRG.9051, MSTRG.10337, MSTRG.18433, and MSTRG.19312). The other high ranking canonical pathway hits, i.e. hits with the lowest p-value, were tRNA charging, leukocyte extravasation signaling, caveolar-mediated endocytosis signaling, and T cell receptor signaling (data not shown).

**Figure 4:**
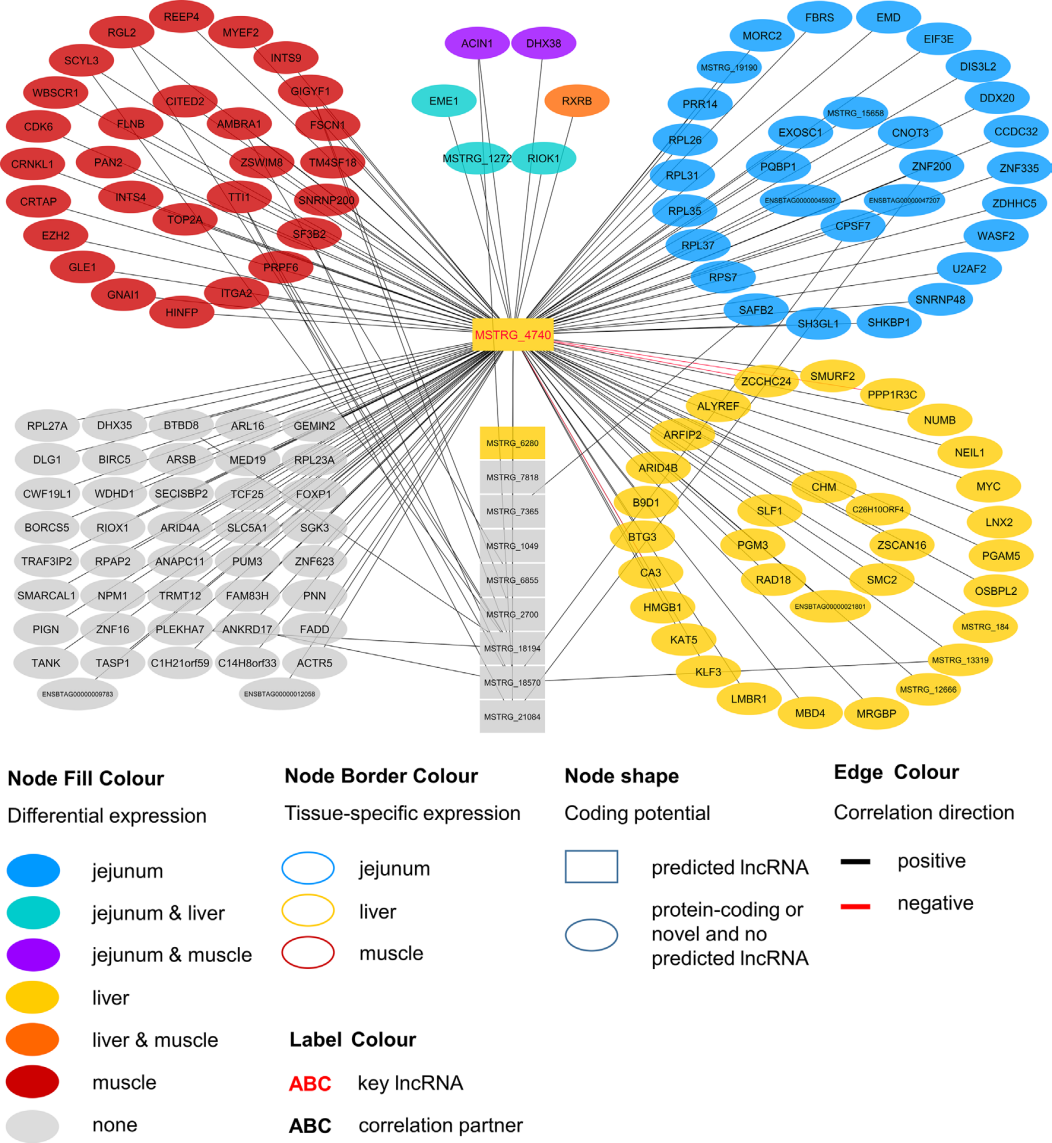
Co-expression network for the novel long non-coding (lnc) RNA MSTRG.4740 with key regulatory potential for metabolic efficiency in cattle and significantly (p < 0.05) correlated genes with a minimal correlation coefficient of |r| > 0.8. Correlations are exclusive for animals with low metabolic efficiency.

**Figure 5:**
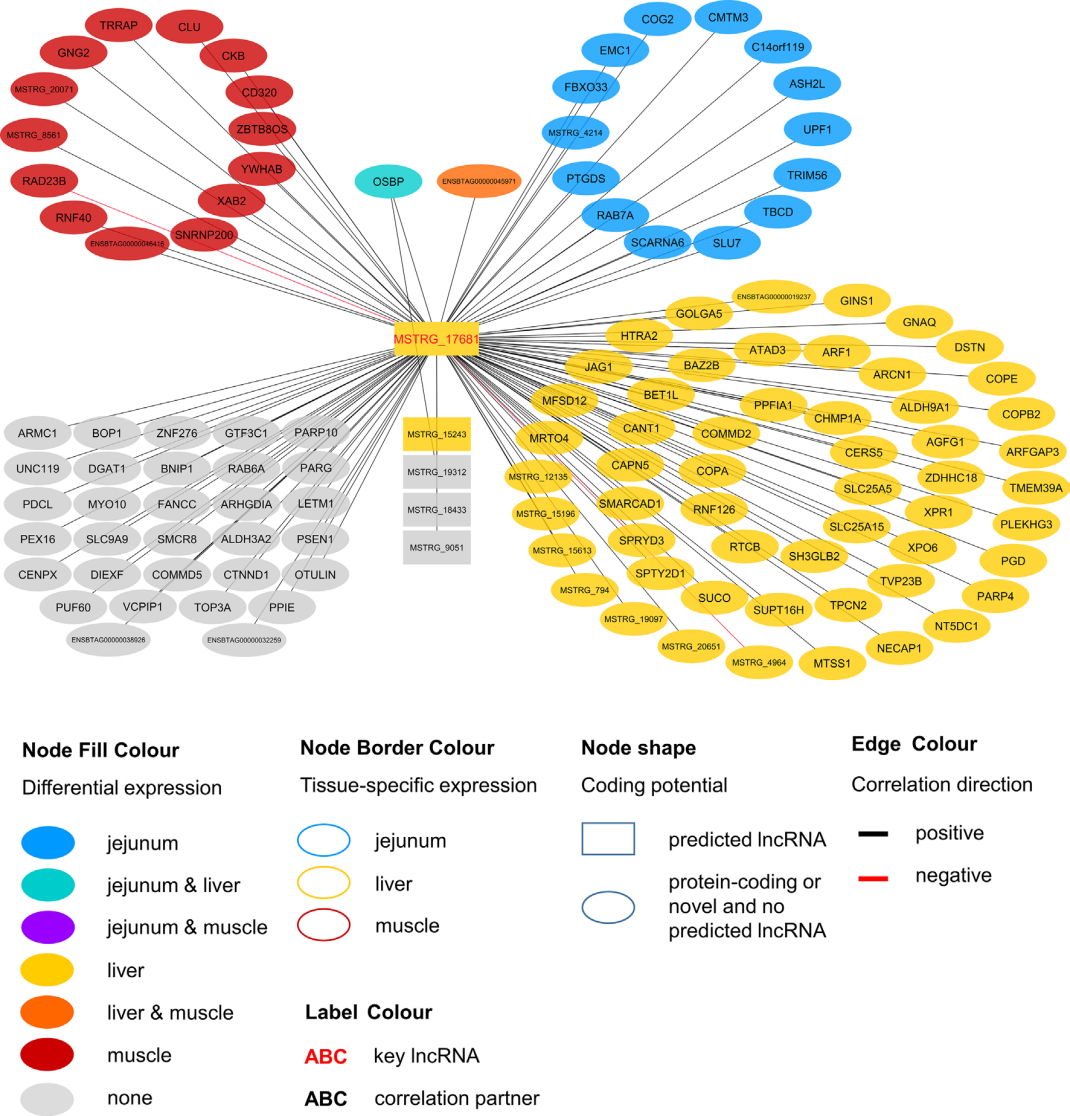
Co-expression network for the novel long non-coding (lnc) RNA MSTRG.17681 with key regulatory potential for metabolic efficiency in cattle and significantly (p < 0.05) correlated genes with a minimal correlation coefficient of |r| > 0.8. Correlations are exclusive for animals with high metabolic efficiency.

**Figure 6:**
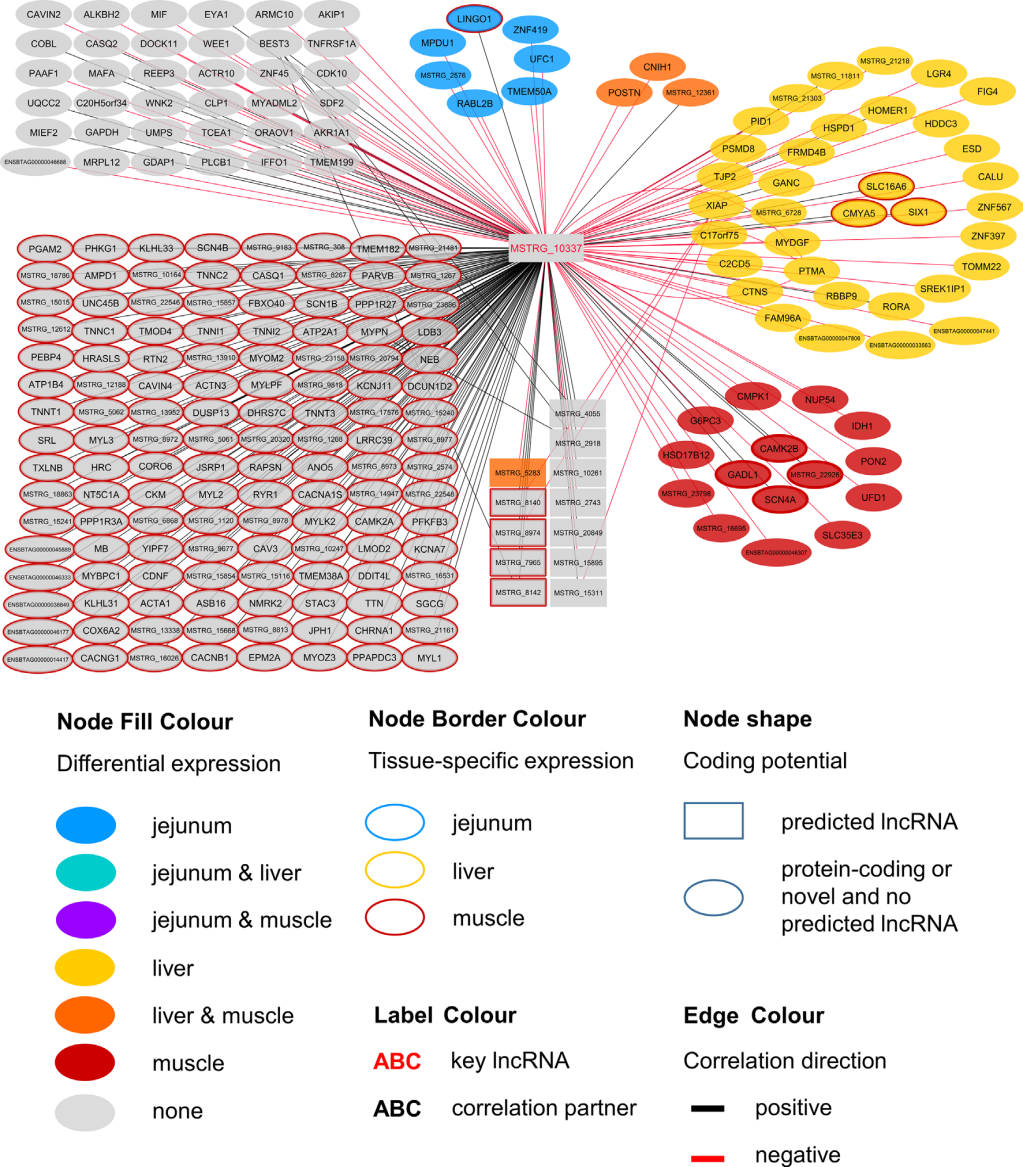
Co-expression network for the novel long non-coding (lnc) RNA MSTRG.10337 with key regulatory potential for metabolic efficiency in cattle and significantly (p < 0.05) correlated genes with a minimal correlation coefficient of |r| > 0.8. Correlations are exclusive for animals with low metabolic efficiency.

Within the eight lncRNAs with a high connectivity in the PCIT analysis, three loci showed distinct pattern in the pathway enrichment analysis suggesting divergent molecular functions. Inspection of the results showed that the enriched canonical pathways for MSTRG.4740, which was differentially expressed in liver (**Figure 4**, **Table 3**, **Supplementary Table 5**), were related to amino acid biosynthesis and metabolism, as well as protein synthesis (**Table 5**). MSTRG.17681 (**Figure 5**, **Supplementary Table 5**) which was also differentially expressed in liver, seemed to act very locally in the coatomer subunit of the coat protein I (COPI) in the caveosome. MSTRG.10337, (**Figure 6**, **Supplementary Table 5**) apparently acts specifically in muscle where it was related to several signaling pathways, most strongly to calcium, protein kinase A, neuronal nitric oxide synthase (nNOS), and RhoA signaling (**Table 5**).

**Table 5:** Top 10 enriched pathways derived from genes and metabolites significantly correlated with key long non-coding RNAs associated with metabolic efficiency

ID	Ingenuity Canonical Pathways	log(p)	Ratio	Molecules
MSTRG.4740	tRNA Charging	5.56E00	8.54E-02	L-valine, L-phenylalanine, L-tryptophan, glycine, L-arginine, L-tyrosine, L-lysine
	EIF2 Signaling	4.13E00	3.83E-02	MYC, RPS7, RPL27A, RPL35, RPL23A, RPL37, RPL26, EIF3E, RPL31
	Glucose and Glucose-1-phosphate Degradation	3.18E00	1.3E-01	D-glucose, PGM3, phosphate
	Tyrosine Biosynthesis IV	2.94E00	2.86E-01	L-phenylalanine, L-tyrosine
	Acetyl-CoA Biosynthesis III (from Citrate)	2.82E00	2.5E-01	phosphate, citric acid
	Glycine Degradation (Creatine Biosynthesis)	2.71E00	2.22E-01	glycine, L-arginine
	Phenylalanine Degradation IV (Mammalian, via Side Chain)	2.68E00	8.82E-02	L-phenylalanine, phenylpyruvic acid, glycine
	Glutathione Biosynthesis	2.53E00	1.82E-01	phosphate, glycine
	Thymine Degradation	2.53E00	1.82E-01	5, 6-dihydrothymine, beta-ureidoisobutyric acid
MSTRG.10337	Calcium Signaling	1.63E01	9.35E-02	TNNT1, CHRNA1, CACNB1, CACNG1, CACNA1S, MYL2, TNNI2, TNNT3,T NNC2, TNNC1, MYL1, ATP2A1, CAMK2A, CASQ1, RYR1, TNNI1, CASQ2, MYL3, ACTA1, CAMK2B
	Protein Kinase A Signaling	7.45E00	3.88E-02	TNNI2, MYL2, MYLPF, MYLK2, PPP1R3A, TTN, MYL1, EPM2A, CAMK2A, PLCB1, RYR1, TNNI1, EYA1, MYL3, CAMK2B, PHKG1
	nNOS Signaling in Skeletal Muscle Cells	6.1E00	1.3E-01	CACNG1, CACNB1, CACNA1S, CHRNA1, RYR1, L-arginine
	Cellular Effects of Sildenafil (Viagra)	6.09E00	6.25E-02	CACNA1S, CACNG1, MYL2, MYLPF, PLCB1, L-arginine, MYL1, MYL3, ACTA1
	RhoA Signaling	4.55E00	5.6E-02	MYL2, MYLPF, MYLK2, TTN, MYL1, MYL3, ACTA1
	Apelin Cardiomyocyte Signaling Pathway	3.7E00	5.00E-02	MYL2, MYLPF, PLCB1, MYL3, MYL1, ATP2A1
	Actin Cytoskeleton Signaling	3.55E00	3.36E-02	MYL2, MYLPF, ACTN3, MYLK2, TTN, ACTA1, MYL3, MYL1
	Regulation of Actin-based Motility by Rho	3.24E00	5.21E-02	MYL2, MYLPF, MYL3, ACTA1, MYL1
	ILK Signaling	3.19E00	3.38E-02	PARVB, MYL2, TNFRSF1A, ACTN3, MYL1, MYL3, ACTA1
	Thrombin Signaling	2.93E00	3.06E-02	CAMK2A, MYL2, MYLPF, PLCB1, MYL1, MYL3, CAMK2B
MSTRG.17681	Caveolar-mediated Endocytosis Signaling	3.56E00	5.48E-02	ARCN1, COPA, COPE, COPB2
	Fatty Acid α-oxidation	2.29E00	8.00E-02	ALDH3A2, ALDH9A1
	Death Receptor Signaling	2.15E00	3.3E-02	PARP10, PARP4, HTRA2
	Histamine Degradation	2.05E00	6.06E-02	ALDH3A2, ALDH9A1
	Oxidative Ethanol Degradation III	2.05E00	6.06E-02	ALDH3A2, ALDH9A1
	G Protein Signaling Mediated by Tubby	2.03E00	5.88E-02	GNG2, GNAQ
	Tryptophan Degradation X (Mammalian, via Tryptamine)	2.00E00	5.71E-02	ALDH3A2, ALDH9A1
	Putrescine Degradation III	2.00E00	5.71E-02	ALDH3A2, ALDH9A1
	Ethanol Degradation IV	1.98E00	5.56E-02	ALDH3A2, ALDH9A1
	NER Pathway	1.96E00	2.8E-02	HIST2H4B, XAB2, RAD23B

## Discussion

A major goal of this study was the identification of lncRNAs that hold a potential key regulatory role in metabolic efficiency, which was roughly defined as the animal's ability to direct the energy adsorbed into protein synthesis and use it for muscle mass accumulation or milk secretion. We integrated phenotypic, metabolomics and transcriptomics data from a cattle F_2_-population (Charolais × Holstein) in a co-expression network approach to mine for lncRNAs with a regulatory role in metabolic processes. By contrasting animals of high and low metabolic efficiency and by including RNAseq data from four key metabolic tissues in a combined analysis, we identified highly connected hub lncRNAs. Finally, we subjected metabolites and genes, whose plasma levels or transcript abundance significantly correlated with expression levels of the specific, highly connected lncRNA, to the integrative approach for metabolomics and transcriptomics data as offered by the cross-platform IPA (Kramer et al., 2014).

### Establishment of a Pipeline Based on Regulatory Impact Factor and Partial Correlation and Information Theory to Establish Co-Expression Networks for Long Non-Coding RNAs and Genes to Predict Their Role in Metabolic Efficiency

Weighted gene co-expression network analysis (WGCNA) (Langfelder and Horvath, 2008) is a frequently applied method to identify co-expression pattern at whole transcriptome level. Recently, Sun et al. (2019) applied this method for mining regulatory signatures of divergent feed efficiency in beef cattle investigating a multi-tissue transcriptome data set. WGCNA has also been used to find hub lncRNAs in a transcriptomic landscape in multiple studies in humans as well as animals (Miao et al., 2016; Tang et al., 2017; Li et al., 2018; Weikard et al., 2018; Wang et al., 2019). To mine for the functional role of lncRNAs of interest via WGCNA, one might select lncRNAs that are strongly correlated with coding neighbor genes (Li et al., 2018) or lncRNAs that were differentially expressed between conditions or phenotypes (Weikard et al., 2018; Wang et al., 2019). The connectivity within a network and the differential wiring between two networks can also serve as a selection criterion (Pellegrina et al., 2017). In our study we present an alternative approach for the selection of lncRNAs of interest, the RIF (Reverter et al., 2010), which has already successfully been applied to transcription factors (TF). In combination with a PCIT (Reverter and Chan, 2008), key regulatory TFs during puberty could be identified in cattle (Cánovas et al., 2014), as well as critical TFs in porcine muscle (Perez-Montarelo et al., 2012). This approach seemed to be particularly applicable for lncRNAs with regard to the expression level as they generally exhibit lower transcript abundance compared with mRNAs (Derrien et al., 2012), as do TFs compared with other coding genes (reviewed by Vaquerizas et al., 2009). We indeed found that only 10% of the unique lncRNAs with a significant RIF-score (n = 240) were also differentially expressed, including three of the eight key hub lncRNAs. LncRNAs were significantly underrepresented in the list of DE loci across all tissues (*Χ*^2^ test, p = 1.2E-06): while they accounted for 14.85% of all loci in the DE analyses, only 11.05% of the DE loci were classified as lncRNAs. In contrast, the other loci accounted for 85.25% of all loci in the DE analyses, but had a share of 88.95% in the total of 2,154 differentially expressed unique loci.

In a recent publication, van Dam et al. (2017) reviewed and highlighted the usefulness of gene co-expression networks for the functional classification of genes and novel loci, such as non-coding elements without any known function. Correspondingly Oliveira et al. (2018) successfully applied a co-expression network concept to identify genes and miRNAs regulating IMF in Nellore steers. Besides the preselection of lncRNAs for co-expression networks, it might be advisable to make a knowledge-based preselection also for other genes to be included instead of simply using all expressed genes. The combination of RNA-Seq results with GWAS hits (gene regions associated with QTL for milk performance traits or RFI) is an acknowledged procedure to integrate multiple layers of knowledge into a prioritized gene set for co-expression network analysis (Schaefer et al., 2018). In our PCIT analysis, we prioritized genes that appeared to be functionally important from the RNA-Seq analysis [DE loci (2,154) or TS loci (930)] and published GWAS data and selected those for our prioritized gene set to create a stronger focus on bovine metabolic efficiency, accepting however that still unknown, yet important elements might be overlooked. When preparing the prioritized gene set, we noted that the key role of liver in metabolic processes was clearly reflected by the by far highest number of DE loci (1,286) between the two metabolic efficiency groups, which was 2.6 fold higher than in jejunum or rumen. For DE loci in the prioritized gene set that was used for the PCIT, we noted that these predominantly (65%) had their highest expression in a different tissue than where they were differently expressed. This underlines that tissue specificity or tissue of highest abundance and DE of loci are indeed different, non-redundant features and that it is recommendable to follow a TS perspective in the beginning of the analysis.

One way to deduce a biological function of lncRNAs is to take a close look at coding genes in their immediate vicinity. This idea has also been implemented in the bioinformatics tool FEELnc for lncRNA prediction and annotation (Wucher et al., 2017), where the potential partner gene is generally assumed to be the closest annotated gene. However, this exclusively focusses on *in-cis* interaction with a narrow frame of impact. However, it has been reported that some lncRNAs execute *in-trans* regulatory tasks by binding directly to distant DNA sites or via RNA-protein interactions (Long et al., 2017) or a direct effect on RNA polymerase II activity (Kornienko et al., 2013).

Another way to infer functionality of unknown genomic elements subsequent to the network construction is to submit correlated coding genes to an enrichment analysis (Chen et al., 2018b), thereby assuming the guilt-by-association principle. Following this approach, we took genes from the prioritized gene set that were correlated with high connectivity lncRNAs of interest. LncRNA partner genes predicted by FEELnc could also be part of the prioritized gene set if they fell into one of the categories (DE, tissue-specificity, QTL-harboring). This was the case for 473 out of 2,741 unique predicted lncRNA interaction partner genes. Thus, 12.6% of the genes that were used as PCIT input (3,754) were very close to or overlapped with a lncRNA.

In addition, we aimed to add a supplementary layer of information to the pathway enrichment analysis and thereby to create further biological depth by using the option to integrate gene expression and metabolic profiles. In a single step this approach facilitates to predict a link between transcriptome activity, the direct functional readout of metabolic activity or physiological status and the functional analysis of lncRNAs. MSTRG.4740, e.g., correlated with plasma levels of 117 metabolites—valuable information that would otherwise be missing from the enrichment analysis. To our knowledge, we here present the first study that integrates metabolomics and transcriptomic data in an enrichment analysis to predict the functional role of lncRNAs.

### Across-Tissue Candidate Long Non-Coding RNAs for Metabolic Efficiency

LncRNAs were defined as hubs when they were connected to at least 100 other nodes in the high or low efficiency PCIT network. Three of the identified eight hub lncRNAs were exemplarily chosen for a more detailed description of their biological functionality predicted with IPA. These lncRNAs—namely MSTRG.4740, MSTRG.10337, and MSTRG.17681—were hubs in gene groups that showed enrichment for transfer RNA (tRNA) charging (p = 2.78E-06) and EIF2 signaling (p = 7.34E-05), calcium signaling (p = 4.98E-17) and nNOS signaling in skeletal muscle cells (p = 7.88E-07), and calveolar-mediated endocytosis signaling (p = 2.77E-04) and fatty acid oxidation (p = 5.13E-03), respectively.

For MSTRG.4740 an encompassing look at the enriched pathways clearly pointed towards amino acid metabolism and protein synthesis. This lncRNA was DE in liver (adjusted p-value (BH) = 9.13E-03, log2FC = 1.70) but displayed highest abundance (average FPKM) in jejunum (10.68) and rumen (8.41) and lowest in muscle (1.66) compared to liver (6.23). The DE status in liver suggested biological relevance there. However, the RIF analysis attributed a significant score to MSTRG.4740 in jejunum. The strongest enrichment was for tRNA charging (p = 2.78E-06), which describes the attachment of amino acids to a tRNA before incorporation into a growing polypeptide. According to IPA, the enrichment of this pathway was due to the correlation of MSTRG.4740 expression level with the blood plasma content of six essential or semi-essential amino acids (L-valine, L-phenylalanine, L-tryptophan, L-arginine, L-tyrosine, L-lysine). No non-essential amino acid showed a significant correlation with this lncRNA. The significantly correlated amino acids play integral roles as regulators of metabolism and key body functions, but cannot or only partially be synthesized by bovine animals themselves. Plasma concentration of essential amino acids depends on uptake from the diet, the balance between protein synthesis and degradation in peripheral tissues as well as on the efficiency of transport processes. The enrichment of the tRNA Charging pathway was not backed up by other components in addition to the indicated amino acids (e.g., charged tRNAs themselves). Thus, we restrict our conclusion and suggest that the lncRNA has a close relationship with (semi-) essential amino acid levels, but rather not to tRNA Charging per se. Widmann et al. (2015) reported no significant correlation between plasma amino acids and RFI at the onset of puberty in bulls in the same resource population. However, in the current study we employed adult animals.

Endogenous metabolism and also supply of amino acid have been demonstrated to limit growth or lactation in pigs, cattle and fish as reviewed by Hou et al. (2016). Furthermore, Doelman et al. (2015) showed that an abomasal infusion with essential amino acids leads to increased protein levels of eIF2α and eIF2Bε in the mammary gland in dairy cows. The authors proclaimed a direct link between the eIF2 factor, which is essential for eukaryotic translation initiation and milk protein yield. Interestingly, we found *eIF2Bε* to be DE [q-value (BH) = 0.022, log2FC = 0.204] in liver and to be one of the genes underlying the significant enrichment of the EIF2 Signaling pathway (p = 7.34E-05), which is tightly linked to protein synthesis. Genes encoding for ribosomal proteins of 40S (*RPS7*) or 60S subunits (e.g. *RPL26*, *RPL31*) were significantly correlated with MSTRG.4740, as well as the before mentioned *eIF2Bε*. EIF2 signaling and subsequently *EIF3E* are required for the correct initiation of mRNA translation (Kimball 1999; Walsh and Mohr, 2014).

Considering the presented correlations of MSTRG.4740 with other genes and plasma metabolites, this hub lncRNA seems to be an excellent example of a potential new key regulator in metabolic efficiency through the modulation of translational processes.

In contrast to MSTRG.4740 that seems to act on the broader forefront of translation, MSTRG.17681 appears to have a rather narrow and more targeted function. The first hit in pathway enrichment was calveolar-mediated endocytosis signaling (p = 2.77E-04). Four genes (*COPA*, *COPE*, *COPB2*, *ARCN1*) belonging to this pathway were highly correlated (|r| > 0.8) with this hub lncRNA. We observed significant DE in the liver of divergently efficient animals for MSTRG.17681 (q-value (BH) = 0.0050, log2FC = 0.766) as well as the respective quartet of genes. *COPA*, *COPE* and *COPB2* are transporters and *ARCN1* encodes the coatomer subunit of the coat protein I (COPI) complex (Tunnacliffe et al., 1996). All genes are allocated to a subunit in the cellular calveolar-mediated endocytosis signaling: the COPI vesicle, which plays a role in intracellular lipid transport (Popoff et al., 2011) and regulates lipid homeostasis (Beller et al., 2008). COPI-vesicle biogenesis is *ARF1*-dependent (Beck et al., 2009), which we found to be DE in liver and to be positively correlated with MSTRG.17681. The *Arf1 GTPase-activating protein* 3 (*ArfGAP3*) that subsequently allows the vesicle to fuse with a target membrane (Beck et al., 2009), was also correlated to MSTRG.17681 and DE in liver.

Considering that COPI-vesicles assist in lipid transport, it seems fitting that we found significant correlations between MSTRG.17681 expression and plasma levels of two saturated fatty acids: caprylate (p = 0.013, r = 0.357) and heptanoate (p = 0.047, r = 0.289). Caprylic acid supplementation in the diet of weaned piglets was observed to lead to a significant increase body weight gain (Marounek et al., 2004). MSTRG.17681 most likely acts predominantly in jejunum, liver, and rumen, where average expression was much higher (31.83, 25.26, and 18.74 FPKM, respectively) compared with the expression in skeletal muscle (3.36 FPKM). We infer that MSTRG.17681 is a key regulator in COPI-vesicle functioning and thereby presumably affects lipid levels.

MSTRG.10337 was the third key hub lncRNA with a distinct prediction of biological function. In the network specific for animals of low metabolic efficiency, MSTRG.10337 was co-expressed with 39 genes that were DE in liver, 4 of which were also DE in muscle. Interestingly, the hub lncRNA MSTRG.10337 correlated with *RORA* (*RAR related orphan receptor A*), which was DE in liver. *RORA* is a transcriptional regulator of genes related to lipid metabolism, e.g. *APOA1*, *APOA5*, *APOC3*, and *PRAPRG* (Vu-Dac et al., 1997; Raspe et al., 2001; Sundvold and Lien, 2001; Lind et al., 2005). Although not meeting the threshold for entering the PCIT network with respect to correlation to MSTRG.10337, we found *APOA1* to be DE in the liver, providing consistency in gene expression and biological interplay with regard to *RORA*. Previously, Krappmann et al. (2012) has attested an association of a *RORC* (*RAR Related Orphan Receptor C*) variant with milk yield, as well as milk fat and protein percentage in our SEGFAM resource population. Furthermore, Zhang et al. (2017) linked both nuclear receptors *RORA* and *RORC* to hepatic lipid and fatty acid metabolism as well as circadian rhythm pathways in a liver-specific depletion experiment in mice.

The most enriched pathways related to MSTRG.10337 are Calcium signaling (p = 4.98E-17) Protein Kinase A (PKA) signaling (p = 3.51E-08), and nNOS signaling in skeletal muscle cells (p = 7.88E-07). These data confirmed findings from an alternative previous network analysis in our resource population, where GWAS results for RFI and metabolomics profiles were merged for bulls in puberty. Widmann et al. (2015) also has identified Protein Kinase A (PKA) signaling and Nitric Oxide signaling to be significantly enriched pathways in IPA analyses.

Calcium signaling, Protein Kinase A (PKA) signaling and nNOS signaling in skeletal muscle cells are in biological interplay. Protein kinases are in charge of nNOS phosphorylation on different serine residues and catalyze the hydroxylation of L-arginine (Fleming, 2008). In turn, L-arginine plasma levels were negatively correlated with expression levels of MSTRG.10337 (p=0.038, r=-0.323) in our study. This would fit an inhibitory role of MSTRG.10337 in metabolic efficiency, because of unfavorable effects of arginine depletion in the diet on milk protein synthesis in dairy cows (Tian et al., 2017). The inhibitory effect is underlined by numerous negative correlations of MSTRG.10337 to genes with DE in liver (e.g. *LGR4*, *FIG4*, *ESD*), muscle (e.g. *PON2*, *IDH1*, *NUP54*) and jejunum (e.g. *LINGO1*, *MPDU1*, *UFC1*), as well as QTL harboring genes (e.g. *GAPDH*, *MAFA*, *MYBPC1*), although the exact mode of operation is unclear. The supplementation of arginine has been reported to reduce body fat deposition, improve muscle gain and improve insulin sensitivity and the metabolic profile (Wu et al., 2009), and its availability in the organism is therefore particularly interesting for beef production. In chicken, L-arginine supplementation enhanced lean muscle growth (Castro et al., 2018). However, protein anabolic effects in muscle via dietary arginine supplementation are controversially discussed in other species (Tang et al., 2011). In addition to Calcium and PKA signaling, a third highly enriched pathway for MSTRG.10337 was nNOS signaling. In terms of gene expression, nNOS is not restricted to neuronal cells but is commonly expressed in skeletal muscle and certain vascular smooth muscle cells as well (Fleming 2008), where it is important for tissue integrity and contractile performance (Percival, 2011). After Ca^2+^-activation, nNOS enzymes produce NO, which affects the autoregulation of blood flow, myocyte differentiation and glucose homeostasis in skeletal muscle cells (Stamler and Meissner, 2001). In a previous study we already suspected a relationship between NO signaling, arginine and growth in cattle (Widmann et al., 2013).

We assume that MSTRG.10337 influences the onset of nNOS activation, because of its correlation to calcium voltage-gated channel genes and *RYR1* (*ryanodine receptor 1*) that encodes a calcium release channel protein (Loy et al., 2011). Co-expression with a large number of muscle specific genes (e.g. *CACNG1, MYLK2, TNNT1, MYL2*) or genes that are DE in muscle (*CAMK2B*) related this hub lncRNA to PKA and nNOS signaling. It might thereby influence phosphorylation, degradation and availability of L-arginine in the muscle cells, but simultaneously perform some regulatory tasks in hepatic lipid metabolism.

## Conclusions

In this study, we were able to identify novel lncRNAs with potential key regulatory function in metabolic efficiency in cattle. Although usually low expression levels of lncRNAs entail difficulties in DE and co-expression analyses, the careful setting of expression thresholds, the use of a-priori knowledge in gene prioritization and the integrated use of RIF metrics and PCIT based co-expression networks have proven to be a valid method for the identification of regulatory hub lncRNAs. The enrichment analysis based on metabolites and gene expression data provided valuable insight into the putative biological functions of yet uncharacterized lncRNAs.

We focused on phenotypic differences and looked at mechanisms or correlations that were exclusive to either metabolic efficiency group. Still, other correlations between lncRNAs and mRNAs might exist simultaneously in both groups, and we propose to take a group transcending approach in a follow-up study. For future work, we suggest to proceed within tissues to get a clearer picture of gene-gene interactions within a tissue, also because we noted that a multi-tissue approach presents its challenges when interpreting pathway enrichment results. The hub lncRNAs, which we identified, can be considered as candidates for further validation studies, *in vitro* or *in vivo*. Kashi et al. (2016) neatly described modern methods to determine where and how lncRNAs act in the cell or organism, such as chromatin isolation by RNA purification (ChIRP) sequencing (Chu et al., 2011).

In conclusion, our study demonstrates that the method we presented is suitable for the identification for key regulatory lncRNAs in a complex phenotype. By carefully adjusting different elements of the procedure, e.g. the tissue under consideration or the choice of priority categories for genes to include in the network analysis, this pipeline allows us to answer targeted biological questions.

## Data Availability Statement

The datasets generated for this study have been submitted to the “Functional Annotation of Animal Genomes” (FAANG) initiative database, accession PRJEB34570, and are also available via the European Nucleotide Archive (ENA).

## Ethics Statement

The animal study was reviewed and approved by Animal care and experimental procedures following the guidelines of the German Law of Animal Protection. The protocols were approved by the Animal Protection Board of the Leibniz Institute for Farm Animal Biology as well as by the Animal Care Committee of the State Mecklenburg-Western Pomerania, Germany (State Office for Agriculture, Food Safety and Fishery; LALLF M-V/ Rostock, Germany, TSD/7221.3-2.1-010/03).

## Author Contributions

WN performed the statistical analyses and investigations, created the visualizations and wrote the original draft. RW and CK performed data collection, generated transcriptomic data, contributed to data analysis and conceptualized and administered the project and supervised WN. AR coded and performed bioinformatics analyses and supervised WN. RB, EA, and HH provided support with sampling and phenotyping of the test animals. All authors contributed to reviewing and editing the manuscript.

## Funding

This study was funded by the German Research Foundation (DFG–grant numbers: KU 771/8-1 and WE 1786/5-1). WN received a scholarship for doctoral candidates from the German Academic Exchange Service (DAAD) and travel funds from the Graduate Academy of the University of Rostock. The publication of this article was funded by the Open Access Fund of the Leibniz Institute for Farm Animal Biology (FBN).

## Conflict of Interest

The authors declare that the research was conducted in the absence of any commercial or financial relationships that could be construed as a potential conflict of interest.
