# Supplementary figures and images for: Biological Network Approach for the Identification of Regulatory Long Non-Coding RNAs Associated With Metabolic Efficiency in Cattle
Source: Front Genet. 2019 Nov 22;10:1130. doi: 10.3389/fgene.2019.01130 (PMC6883949; doi:10.3389/fgene.2019.01130)

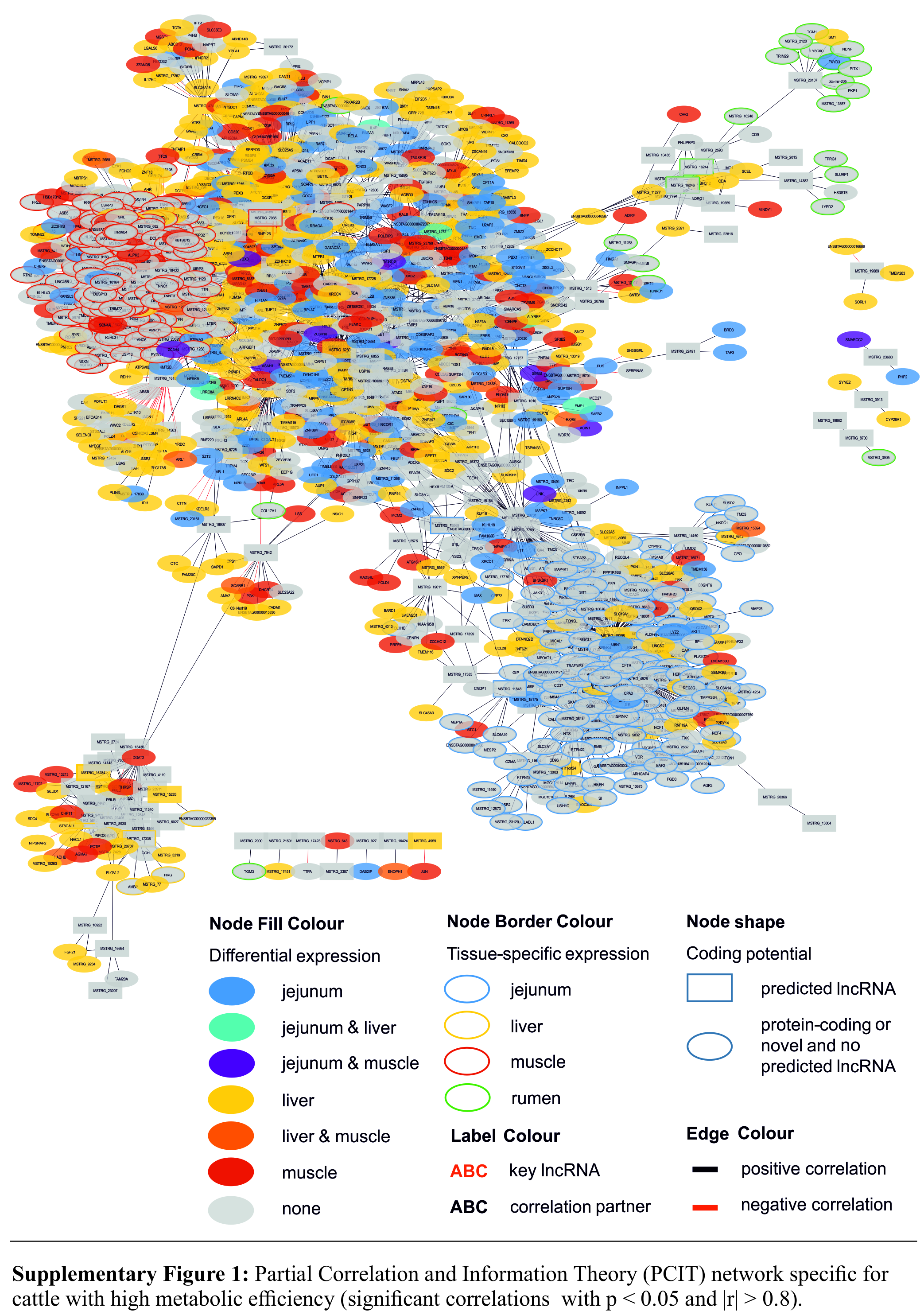

Supplement: Supplementary file 1 [file Image_1.tif]

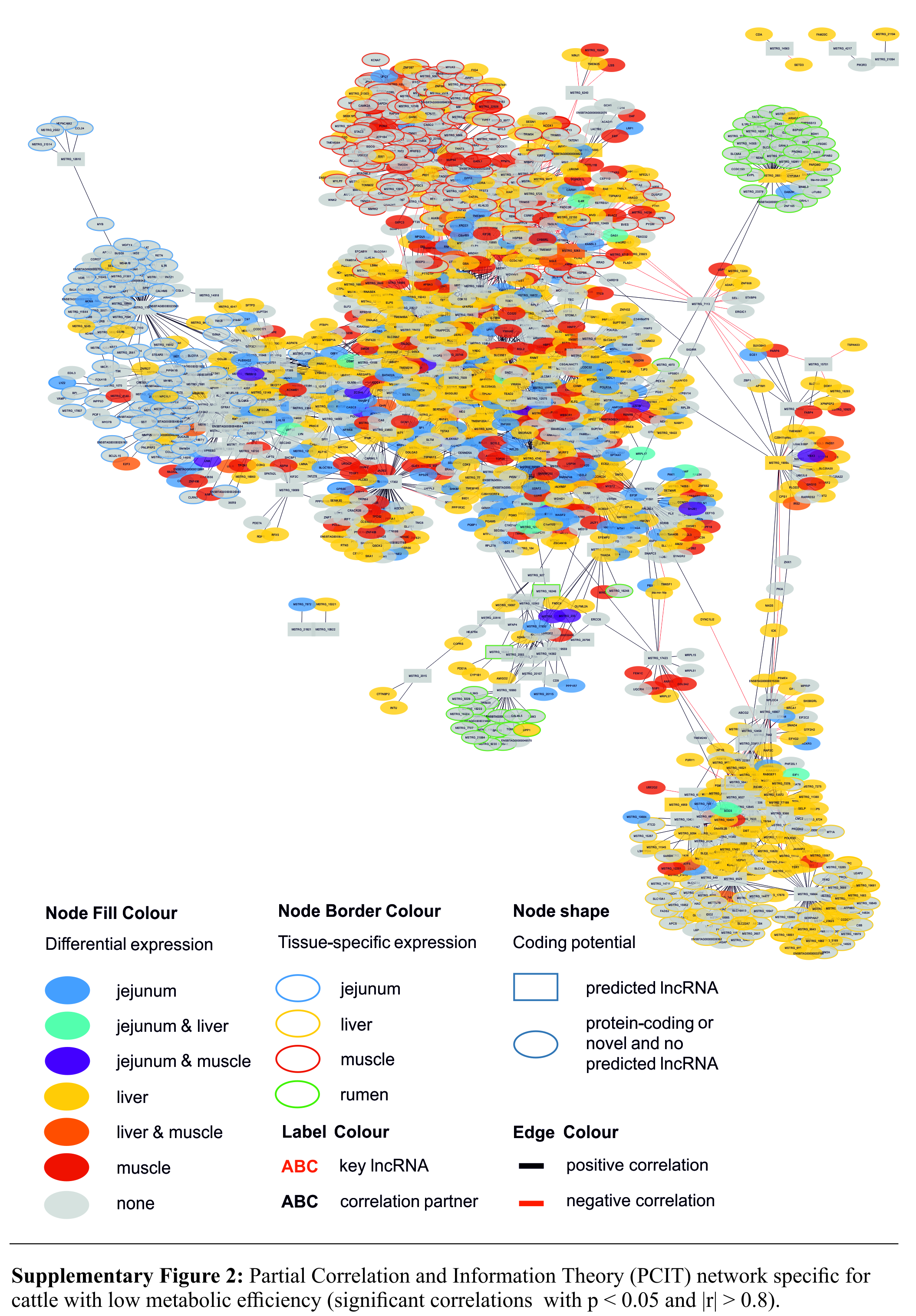

Supplement: Supplementary file 2 [file Image_2.tif]
